# Benign Recurrent Vertigo: The Course of Vertigo Attacks Compared to Patients With Menière's Disease and Vestibular Migraine

**DOI:** 10.3389/fneur.2022.817812

**Published:** 2022-03-02

**Authors:** Roeland B. van Leeuwen, Carla Colijn, Babette F. van Esch, Tjard R. Schermer

**Affiliations:** ^1^Apeldoorn Dizziness Center, Gelre Hospitals, Apeldoorn, Netherlands; ^2^Department of Otorhinolaryngology-Head and Neck Surgery, Leiden University Medical Centre, Leiden, Netherlands; ^3^Department of Primary and Community Care, Radboud Institute for Health Sciences, Radboud University Medical Center, Nijmegen, Netherlands

**Keywords:** benign recurrent vertigo, vestibular migraine, Menière's disease, attacks, prognosis, mental health limitations, medication

## Abstract

**Objective:**

To explore the course of vertigo attacks in patients with benign recurrent vertigo (BRV) as compared to patients with Menière's disease (MD) and vestibular migraine (VM).

**Study design:**

Prospective cohort study.

**Setting:**

Tertiary referral center.

**Patients:**

Adult patients who visited the Apeldoorn Dizziness Center between January 2015 and November 2016 and who were diagnosed with BRV, VM or MD. During 3 years participants were contacted every 6 months by telephone to complete a study-specific questionnaire.

**Main Outcome Measures:**

Vertigo attack frequency, use of medication, and Hospital Anxiety and Depression Scale (HADS).

**Results:**

The study population (*n* = 121) consisted of 44 patients with BRV, 34 with VM, and 43 with MD. For the total follow-up period no statistically significant differences between the three diagnosis groups were observed for being attack-free in the past 6 months: OR = 0.86 (95% CI 0.34–2.17; *p* = 0.745) for VM and OR = 1.06 (95% CI 0.44–2.51; *p* = 0.902) for MD, compared to BRV. Overall, 19 patients (43.2%) with BRV, 13 (38.2%) with VM, and 35 (81.0%) with MD used medication to prevent vertigo attacks at any point during their 3-year follow-up. Throughout the observation period patients with MD showed an average of 3.37 points (95% CI 0.68–6.07; *p* = 0.014) higher HADS scores relative to patients with BRV.

**Conclusion:**

The course of vertigo attacks was rather favorable in the three groups, as 67–70% of the patients were free of vertigo attacks after 3 years of follow-up. The course of disease in patients with BRV was not distinctive from patients with MD and VM. We assume that BRV is a mild or incomplete variant of VM and MD, rather than a separate disease entity with distinct pathognomonic features.

## Introduction

The clinical syndrome “benign recurrent vertigo” (BRV) (or “recurrent vestibulopathy”) was first described by Slater in 1979 and by Leliever and Barber in 1981 (1, 2). The syndrome describes multiple episodes of vertigo lasting 5 min−24 h accompanied by nausea and vomiting. No signs of clinical neurological or auditory abnormalities are manifested and head movements are not provoking vertigo attacks. According to the Committee on Hearing and Equilibrium guidelines, benign recurrent vertigo is considered to be a syndrome with recurrent vertigo attacks without hearing loss, tinnitus or aural fullness (3). So far, the Bárány Society has not formulated criteria, so a formal approval of this syndrome is not yet established.

The etiology of BRV remains unknown. Some claim the involvement of vestibular migraine (VM), while others believe that Menière's disease (MD) is related to this vertigo syndrome (4–6). The criteria formulated for “probable” VM (pVM) closely resemble the criteria applied in BRV (7). BRV is frequently diagnosed and the majority of the cases show spontaneous resolution of vertigo attacks (Table 1) (8–11).

**Table 1:** Previous studies and the current study on vertigo attacks in patients with benign recurrent vertigo.

	***n***	**Follow-up**	**Free of attacks**	**Amended diagnosis**		
**References**				**Menière's disease**	**Vestibular migraine**	**Other**
Leliever and Barber (2)	63	3.5 years	70%	10%	–	2%
Rutka and Barber (11)	61	8.5 years	63%	14%	–	–
Van Leeuwen and Bruintjes (8)	89	31 months	62%	1%	2%	–
Lee et al. (10)	98	63 months	82%	4%	2%	–
Pan et al. (9)	56	32 months	24%	–	10%	–
Current study	44	36 months	71%	None	None	None

In 2017, we reported clinical characteristics of three groups of patients {i.e., BRV, VM [definite VM (dVM) and pVM], and MD} (12). The aim of this previous study was to systematically compare the clinical characteristics of patients with BRV, VM, and MD and to assess whether clinical symptoms existed that were unique to BRV thus discriminating it from VM and MD.

The aim of the current study in the same cohort of patients is to identify whether vertigo attacks, vertigo medication use and mental health limitations differ between patients diagnosed with BRV, VM or MD during 3 years of follow-up.

## Methods

We performed a prospective cohort study in patients recruited at the Apeldoorn Dizziness Center (ADC) between January 2015 and November 2016; the follow-up period ended December 2019. The ADC is a tertiary center providing specialized care for patients suffering from dizziness. Four groups of patients (BRV, dVM, pVM, and MD) were selected according to the criteria for the different diagnosis groups that applied in 2015 (1, 3, 7). For the methods for selection of the groups we refer to our previous study (12). In the current follow-up study of the cohort the dVM and pVM groups were combined in a single VM group.

### Outcomes and Measurements

We performed follow-up assessments based on a comprehensive telephone survey at 3, 6, 12, 18, 24, 30, and 36 months after the baseline assessment. If we suspected a change of diagnosis during the follow-up, additional diagnostic testing was performed if required after consultation of an ENT surgeon or a neurologist. All patients diagnosed with VM or MD received standard therapy if necessary.

The course of vertigo attacks was assessed using the following outcomes: frequency of vertigo attacks (number of attacks in the past 6 months), additional clinical features such as ear-related symptoms and headache, and need for (additional) treatment: preventive medication or intratympanic injections.

Patients completed the Hospital Anxiety and Depression Scale (HADS) (13) at every follow-up assessment in order to determine the levels of anxiety and depression which might be related to the vertigo attacks. The HADS is a self-rating questionnaire and is considered to measure psychological distress as a screening diagnostic rather than detecting psychiatric comorbidity (14). It consists of an anxiety subscale and a depression subscale, both with seven items. A higher HADS score means higher levels of anxiety or depression. A total score ≥ 7 for one of the subscales is considered an indication for psychological distress (14). In order to look at the clinical relevance of change in HADS score over time in individual patients, a minimal clinically important difference (MCID) was defined as a ≥2.7 point reduction in HADS score between baseline and the 3-year follow-up measurement (2.7 was half the standard deviation of the baseline HADS score in the total study sample) (12, 15).

### Statistical Analysis

Student t, Mann-Whitney U and Chi-square tests were used to test differences in demographic and clinical characteristics between the diagnosis groups at baseline (12). To analyze the course of vertigo attacks throughout the 3-year observation period, the attack frequency that patients reported during the semi-annual follow-up contacts was dichotomized as either “attack-free” or “not attack-free” in the past 6 months. The probability of being attack-free was compared between the three diagnosis groups using generalized estimating equations (GEE) models with binomial distribution, logit link function and an unstructured correlation matrix. Results are expressed as odds ratios (ORs) and their 95% confidence intervals (95% CIs), with the BRV group serving as the reference group (i.e., OR = 1). The GEE models included interaction terms for the follow-up contact ^*^ diagnosis group. Age and sex were included as covariates to correct for baseline differences in demographic characteristics between the diagnosis groups.

The semi-annually measured HADS scores were also analyzed using GEE models, with normal distribution, identity link function, unstructured correlation matrix, follow-up contact ^*^ diagnosis group interaction terms, and age and sex included as covariates, again with the BRV group serving as reference. To analyze if patients who reported to be attack-free at the 3-year follow-up measurement showed different HADS scores compared to those who still experienced vertigo attacks, linear regression analysis was applied for each of the diagnosis groups separately. In these regression models the estimate for the HADS score at 3-year follow-up was adjusted for baseline HADS score, age, and sex. Proportions of patients with a MCID on the HADS score were compared between attack-free and not attack-free patients within the three diagnosis groups and tested using Fisher's exact tests.

All statistical analyses were performed using SPSS software (Version 25.0, released 2017. IBM SPSS Statistics for Windows, Armonk, NY). A *p*-value < 0.05 was considered to be statistically significant.

### Ethical Considerations

The study was designed and conducted in compliance with the Helsinki Declaration. Approval of the local institutional review board was obtained and all data was deidentified. All patients gave written informed consent before entering the study.

## Results

### Study Population

**Figure 1:**
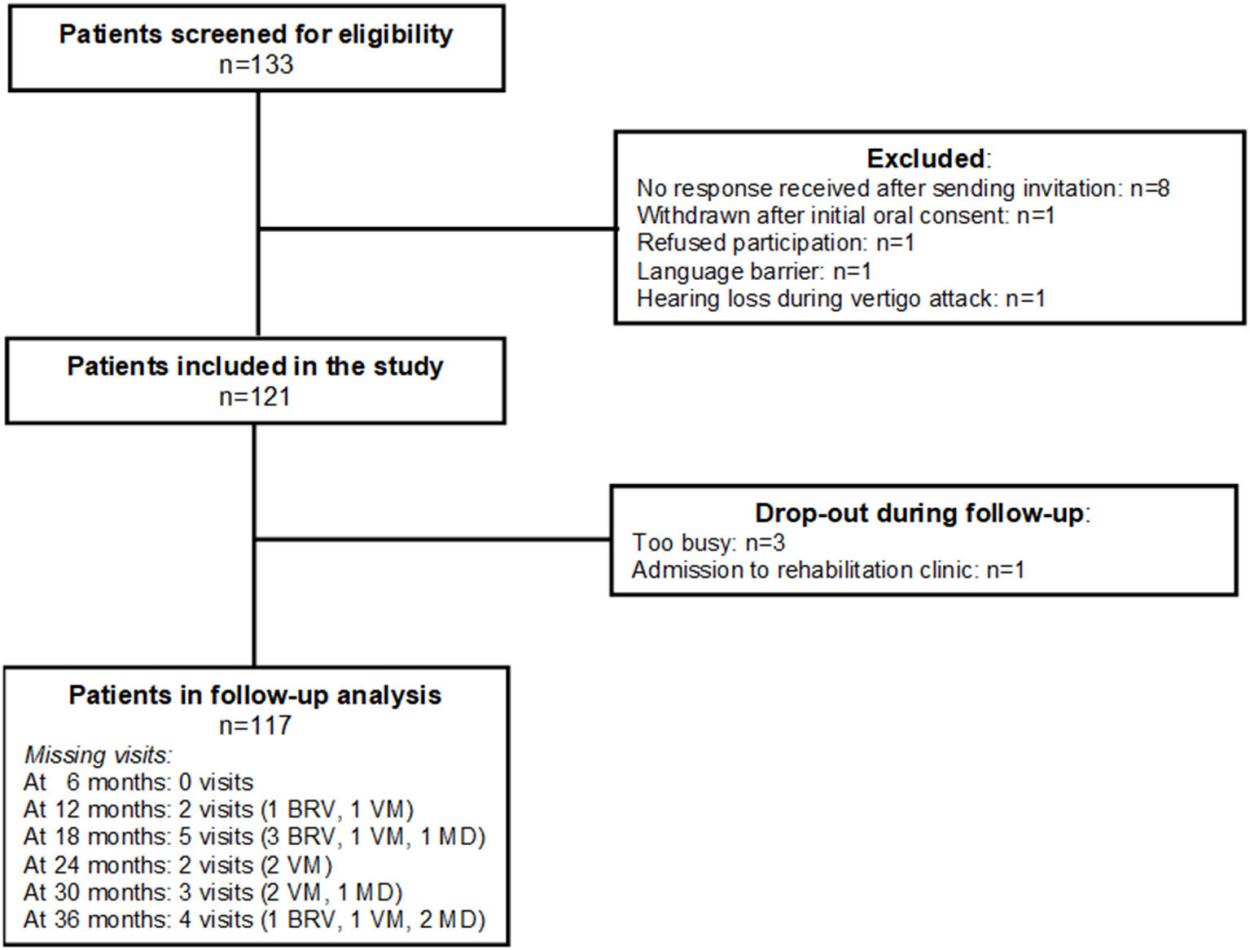
Inclusion and follow-up of the study population. BRV, benign recurrent vertigo; VD, vestibular migraine; MD, Menière's disease.

The study population (*n* = 121) consisted of 44 patients with BVR, 34 with VM, and 43 with MD. One of the patients from the original study cohort (*n* = 122) refused to participate in this follow-up study. Figure 1 shows that during the 3-year follow-up period 4 patients (1 BRV, 2 VM, 1 MD) dropped out of the study. Table 2 shows a summary of the baseline characteristics for the three diagnosis groups.

**Table 2:** Demographic and clinical characteristics of the three diagnosis groups at initial presentation.

	**Benign recurrent vertigo** **(*n* = 44)**		**Vestibular migraine** **(*n* = 34)**		**Menière's disease** **(*n* = 43)**	
	***n***	**(%)**	***n***	**(%)**	***n***	**(%)**
Sex, *n* (%)						
Male	20	(44)	9	(27)	28	(65)
Female	24	(56)	25	(73)	15	(35)
Age [in yr; mean (SD)]	59.8	(11.6)	52.9	(14.1)	53.2	(14.6)
Age of onset symptoms [in yr; mean (SD)]	52.6	(13.9)	45.0	(15.8)	46.6	(14.1)
Family history of migraine	12	(27.3)	16	(47.1)	7	(16.3)
Family history of Menière's disease	2	(4.5)	2	(5.9)	7	(16.3)
Vertigo attack frequency last 6 months:						
<2 attacks	7	(15.9)	6	(17.6)	4	(9.3)
2–10 attacks	31	(70.5)	22	(64.7)	20	(46.5)
≥10 attacks	6	(13.6)	6	(17.6)	19	(44.2)
Duration of vertigo attacks:						
<24 h	32	(72.7)	25	(74.5)	41	(95.3)
≥24 h	12	(27.3)	9	(26.5)	2	(4.7)
**Additional symptoms during attacks:**						
Vegetative symptoms:						
Nausea	37	(84.1)	33	(97.1)	39	(90.7)
Vomiting	28	(63.6)	20	(58.8)	32	(74.4)
Auditory symptoms:						
Hearing loss	5	(13.2)	3	(9.4)	29	(80.6)
Tinnitus	14	(31.8)	16	(47.1)	36	(83.7)
Aural fullness	8	(18.2)	12	(35.3)	29	(67.4)
Migraine-related symptoms:						
Aura	5	(11.4)	11	(32.4)	10	(23.3)
Photophobia	10	(22.7)	18	(52.9)	17	(39.5)
Phonophobia	10	(22.7)	15	(44.1)	23	(53.5)
Contributing factors:						
Stress	18	(40.9)	26	(76.5)	27	(62.8)
Menstrual cycle	2	(4.5)	4	(11.8)	1	(2.3)
Fatigue	14	(31.8)	24	(70.6)	21	(48.8)
Head movement	15	(34.1)	15	(44.1)	20	(46.5)
Alcohol	1	(2.3)	2	(5.9)	4	(9.3)

*Figures are numbers (%) unless stated otherwise*.

### Medication Use

**Figure 2:**
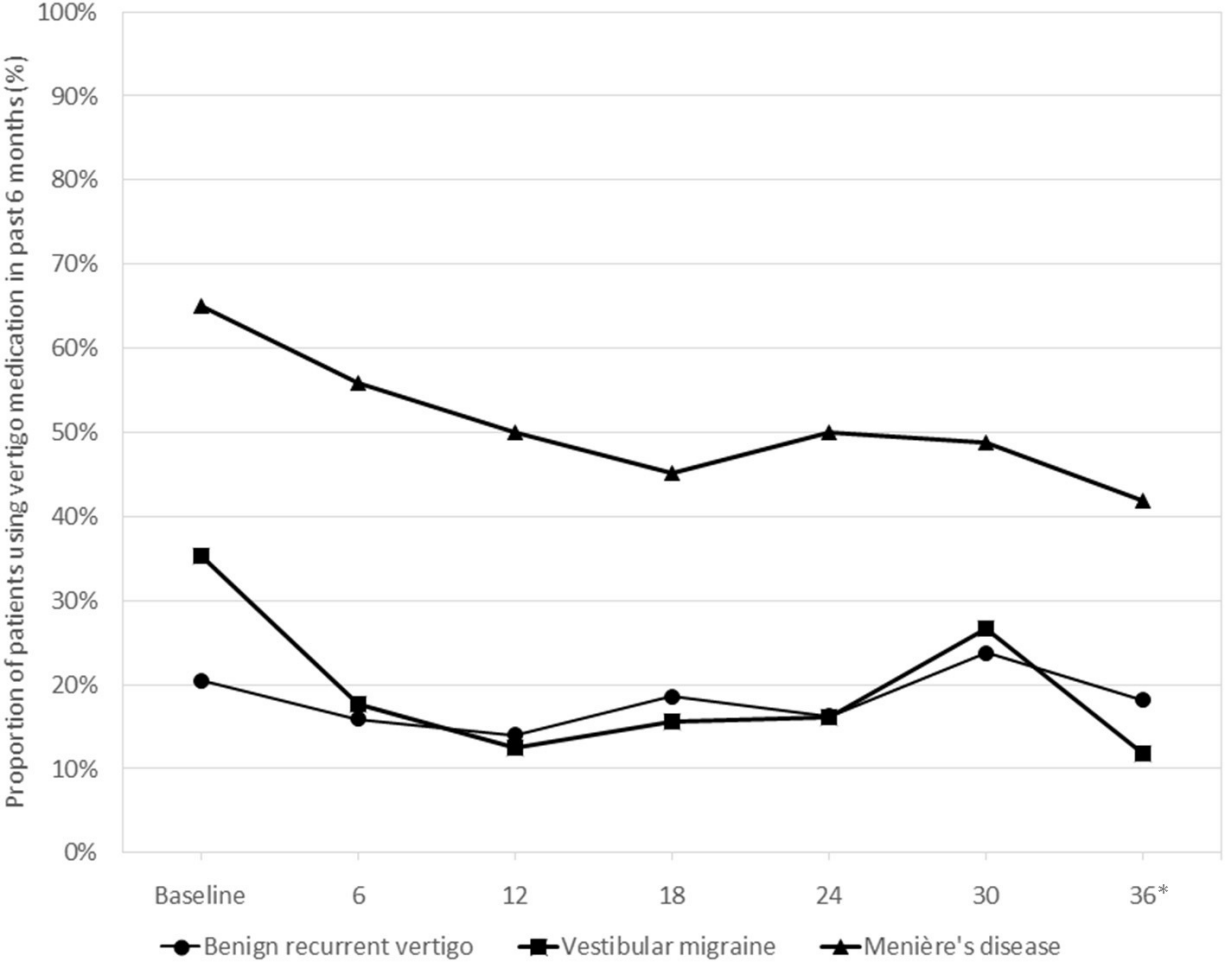
Use of vertigo medication during the 3-year follow-up period in the three diagnosis groups*. *Medication use in the past 6 months was tested at 36 months, the difference between the groups being statistically significant (Chi-square test, *p* < 0.001).

Overall, 19 patients (43.2%) with BRV, 13 (38.2%) with VM, and 35 (81.0%) with MD used medication to prevent vertigo attacks at any point during their 3-year follow-up. The difference in vertigo medication use between the three diagnosis groups was consistent over time, with MD patients consistently reporting more medication use and the other two diagnosis groups showing very similar proportions using medication (Figure 2). At 36 months, the difference in medication use between the groups was statistically significant (Chi-square test, *p* < 0.001). Ten patients (23.3%) with MD received one or more intratympanic injections during follow-up.

### Vertigo Attacks

**Figure 3:**
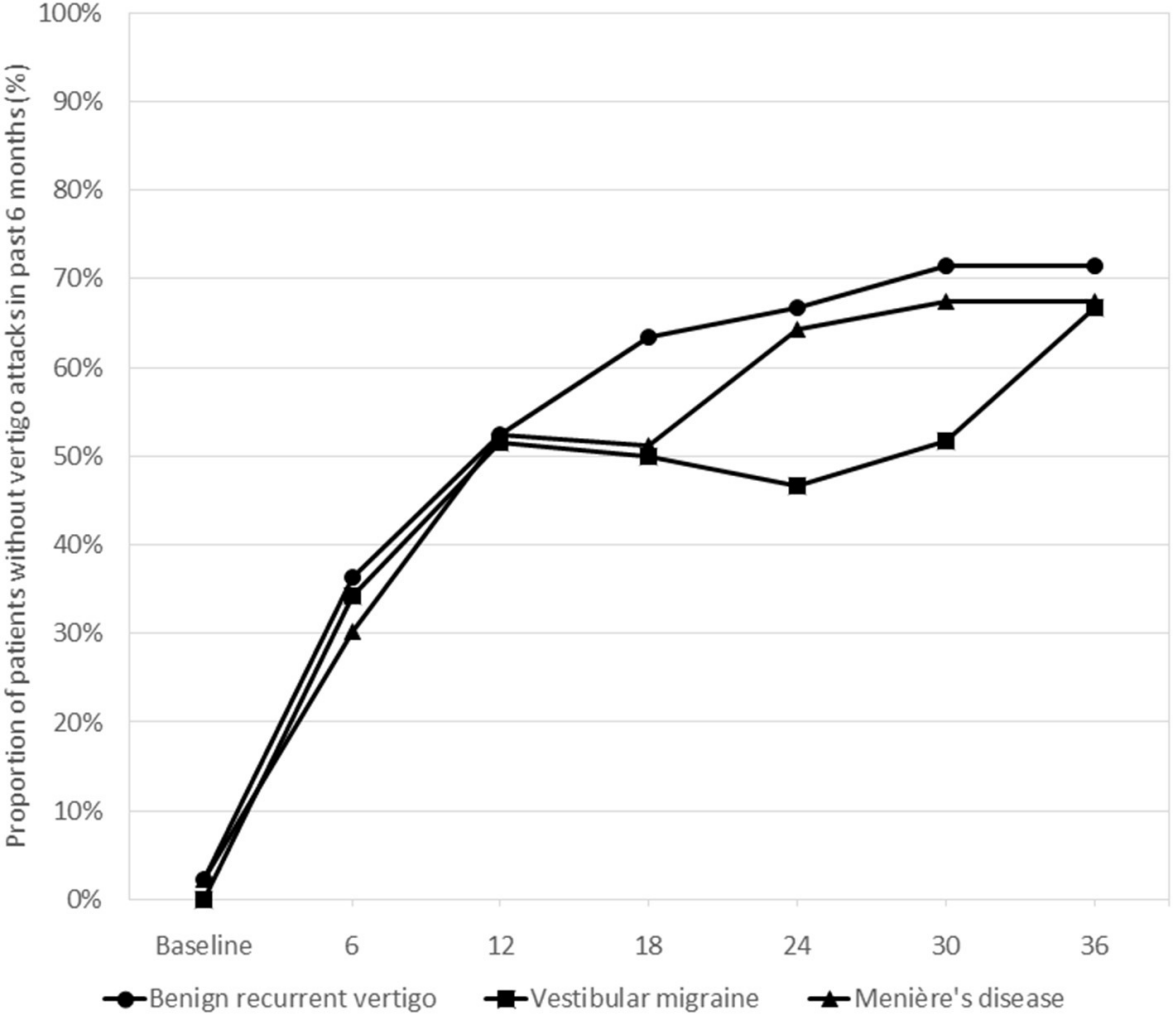
Proportions of patients without vertigo attacks during the past 6 months as reported by the patients in the three diagnosis groups at the consecutive follow-up visits. Differences between the groups were statistically tested using generalized estimating equations models. Overall, there were no statistically significant differences between the groups for the follow-up period as a whole.

Figure 3 shows the probability of being attack-free during the semi-annual follow-up contacts for the three diagnosis groups. At 36 months 71.4% of the BRV, 66.7% of the VM, and 67.5% of the MD patients were attack-free (Chi-square test, *p* = 0.909). For the total follow-up period no statistically significant differences between the three diagnosis groups were observed for being attack-free in the past 6 months: OR = 0.86 (95% CI 0.34–2.17; *p* = 0.745) for VM and OR = 1.06 (95% CI 0.44–2.51; *p* = 0.902) for MD, compared to BRV.

In none of the three diagnosis groups, the patients who reported to be attack-free at 36 months showed a different use of vertigo medication in the past 3 years compared to those who still reported to have attacks (Chi-square test, *p* ≥ 0.24 in all three groups).

### HADS Scores

**Figure 4:**
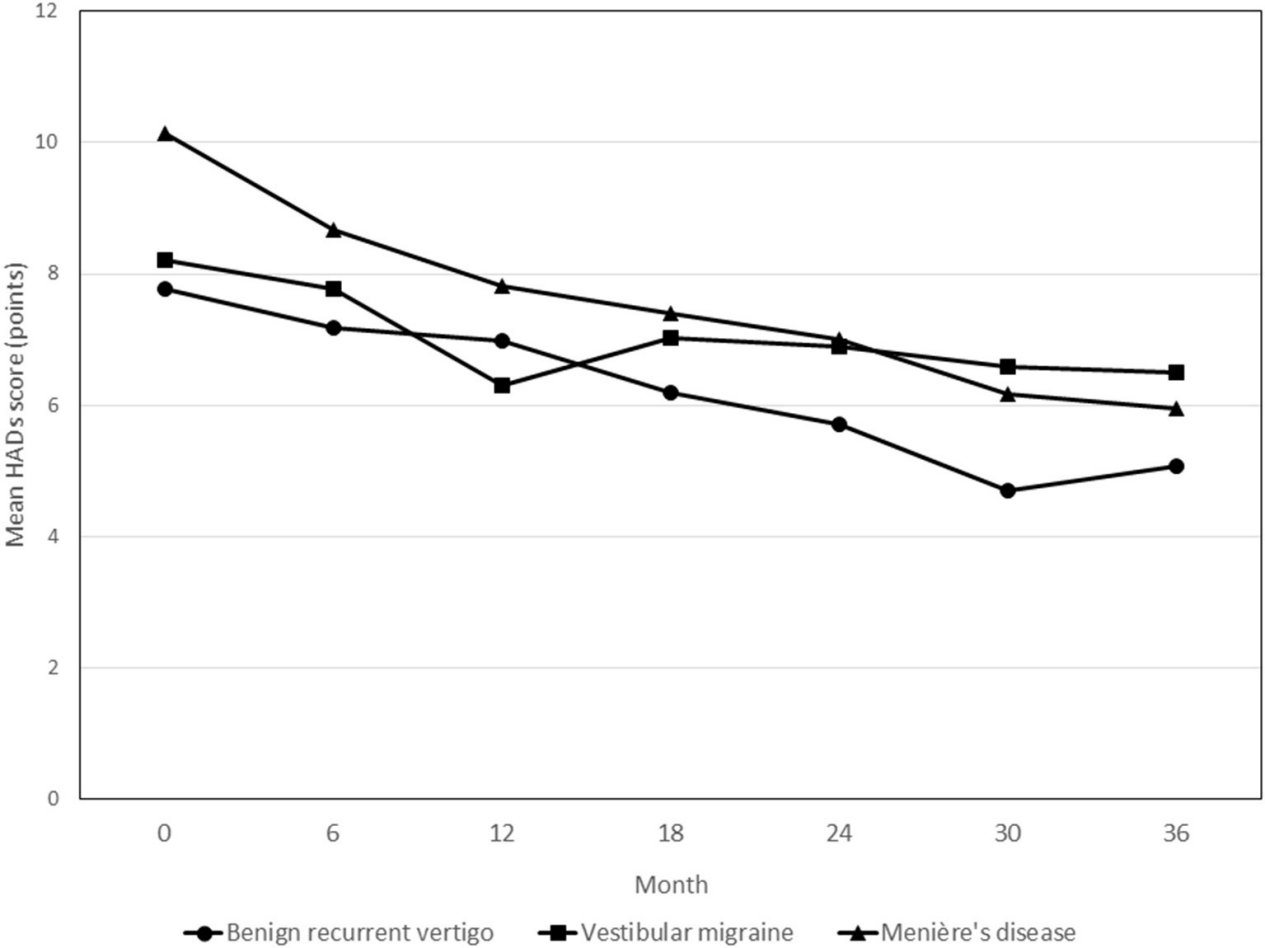
Course of HADS scores during the 3-year follow-up period in patients with benign recurrent vertigo, vestibular migraine and Menière's disease. None of the follow-up contact * diagnosis group interaction terms showed statistically significant differences in HADS scores. HADS, Hospital Anxiety and Depression Scales.

Figure 4 shows the course of HADS scores over time in the three diagnosis groups. Throughout the observation period, patients with MD showed an average of 3.37 points (95% CI 0.68–6.07; *p* = 0.014) higher HADS scores relative to patients with BRV. Overall, the patients in the VM group did not show different HADS scores compared to the BRV patients (0.67 points (95% CI −1.82 to 3.16; *p* = 0.597).

In the BRV and MD groups, patients who reported to be vertigo attack-free at 36 months showed statistically significant changes in HADS scores from baseline at the final measurement compared to patients who still reported to have had attacks in the past 6 months [−4.0 points (95% CI −6.9 to −1.2; *p* = 0.007) and −3.7 points (95% CI −7.0 to −0.4; *p* = 0.028) for BRV and MD, respectively]. In the VM group, no such statistically significant difference was observed [3.2 points (95% CI −0.8 to 7.2; *p* = 0.114)].

BRV patients who were attack-free after 3 years showed a statistically significant higher proportion of patients with MCID on the HADS score compared to patients who still experienced vertigo attacks (60 vs. 18%, *p* = 0.032). No statistically significant differences in the proportion with MCID between patients with and without attacks were observed for the VM and MD groups (*p* = 0.108 and *p* = 0.087, respectively).

### Diagnostic Amendments

In the BRV group, there were no diagnostic amendments to MD or VM during the 3 year follow-up period (Table 1).

## Discussion

While our first study focused on the clinical characteristics of BRV compared to VM and MD (12), the aim of the current study was to investigate the course of vertigo attacks, vertigo medication use and mental health limitations in patients with BRV compared to patients diagnosed with VM and MD. The course of vertigo attacks was favorable in all three groups. After 3 years of follow-up, 67–70% of the patients was free of vertigo attacks. In the BRV group, there were no diagnostic amendments to MD or VM during follow-up. The use of medication or intratympanic injections was not related to the course of vertigo attacks. The HADS showed a significant decrease in patients with MD, but this was less clear for patients with VM or BRV.

Like in most other studies, our BRV patients showed a favorable course with a strong reduction of frequency of attacks (Table 1). As a result, in patients with BRV, the course of the disease does not differ from the course in VM and MD. This is in contrast with the unfavorable course in BRV as reported in the study of Pan et al. (9).

Although changes to the diagnosis were frequently made in other studies (especially in patients initially diagnosed with MD), in none of the patients in our sample clinical symptoms evolved resulting in a change of the diagnosis. One possible explanation for this could be that we were very strict in the application of the criteria for the three diagnoses. We are not aware of any studies that have investigated the impact of medication and/or intratympanic injections on disease prognosis. The difference in the concurrent use of medication between the patients with a favorable course in terms of vertigo attacks and patients with an unfavorable course was not statistically significant in any of the three groups.

The course of the HADS scores showed a gradual but moderate decrease in the BRV group. The most significant decrease was observed in the MD group, in which the mean baseline HADS score was also the highest.

There is ongoing debate about the question to what extent BRV can be described as a separate clinical entity. In our tertiary dizziness center (1,200 new patients per year) we see a large group of patients with severe vertigo attacks, which often last several hours, without headaches or ear symptoms. These patients do not meet the criteria for VM or MD.

A reasonably large group of patients with sudden or occasional vestibular symptoms cannot be diagnosed in accordance with the current criteria of the Bárány Society. In a recent publication by Dlugaiczyk et al., this heterogenous group of patients is defined as “recurrent vestibular symptoms not otherwise specified” (RVS-NOS) (16). The difference with our definition of BRV is that the nature, duration and accompanying symptoms are not debilitating. Moreover, they can be differentiated from well-known diagnoses such as MD, VM, Benign Paroxysmal Positional Vertigo (BPPV) and vestibular paroxysmia. As such, BRV can be considered as a sub-group within RVS-NOS. Dlugaiczyk et al. (16) concluded that RVS-NOS is more likely to be composed of several disorders including a spectrum of mild or incomplete variants of known vestibular disorders, such as VM and MD, rather than being a separate disease entity with distinct pathognomonic features. We support this conclusion. In our opinion, BPV is probably not a separate entity, but it is closely linked to VM and—to a lesser degree—to MD.

A strength of our study is the fact that we have little missing data, as only 4 patients dropped out and 98% of the planned follow-up contacts took place. Moreover, our study has a reasonably long follow-up, with frequent repetition of measurements. At the same time, our study has a number of limitations. First, it was conducted in a tertiary expertise center, which might induce the selection of patients with more or more severe complaints. Second, the clinical course after the visit to our center can be influenced by the frequent use of medication or intratympanic injections. Third, the three study groups are not very large, primarily due to our strict application of the criteria for the three diagnoses. Last, the follow-up was conducted through telephone interviews and not through actual visits to our center, and therefore we did not systematically perform audiograms to establish a subclinical change to MD in the BRV and VM groups.

The practical significance of our findings is that, in our view, patients who are diagnosed with BRV can be informed that their prognosis in terms of vertigo attacks is rather favorable, and also that it is unlikely that it will further develop into MD.

We conclude that BRV has a favorable course when it comes to the frequency of attacks in the course of 3 years, as 71% of the patients were free of vertigo attacks. Nonetheless, there is a parallel in the course of disease similar to VM and MD and no significant differences could be demonstrated. We assume that BRV is an incomplete variant of VM or MD—not a separate entity with distinct pathognomonic features.

## Data Availability Statement

The raw data supporting the conclusions of this article will be made available by the authors on request, without undue reservation.

## Ethics Statement

The study was reviewed and approved by the Gelre Hospitals Institutional Review Board. All participants provided their written informed consent to participate in this study.

## Author Contributions

RL and BE initiated the study. RL, BE, and CC wrote the study protocol. CC and BE were responsible for the study logistics, data collection, and critically reviewed the contents of the draft manuscript. TS and CC wrote the plan for data cleaning and analysis and performed the statistical analyses. RL and TS wrote the draft version of the manuscript. RL is the guarantor of the study. All authors have seen and confirmed the final version of the manuscript that was submitted for publication.

## Funding

The study was funded by the Apeldoorn Dizziness Center, Gelre Hospitals, Apeldoorn, Netherlands.

## Conflict of Interest

The authors declare that the research was conducted in the absence of any commercial or financial relationships that could be construed as a potential conflict of interest.

## Publisher's Note

All claims expressed in this article are solely those of the authors and do not necessarily represent those of their affiliated organizations, or those of the publisher, the editors and the reviewers. Any product that may be evaluated in this article, or claim that may be made by its manufacturer, is not guaranteed or endorsed by the publisher.
